# THE INFLUENCE OF NURSING HOME CONTEXT ON IMPLEMENTATION: EARLY FINDINGS

**DOI:** 10.1093/geroni/igac059.1236

**Published:** 2022-12-20

**Authors:** Carole Estabrooks, Anna Beeber, Amy Vogelsmeier

## Abstract

Evidence suggests that organizational context, including context of care in nursing homes (NHs) is associated with implementation and improvement success and that optimized context contributes to improved resident and staff outcomes. However, evidence on mechanisms of these influences is scarce. The longitudinal Translating Research in Elder Care program (2007 to present) is our data source. This symposium reports findings of a secondary analysis of longitudinal organizational structural and contextual, staff worklife, and resident care quality data collected in nursing homes from 2007-2021, in a cohort of 94 Canadian nursing homes. Our data include continuously collected administrative data (RAI-MDS 2.0), 5 waves of primary surveys from multiple care providers in NHs (15,000 cases to date), extensive qualitative case study data, and data from two large scale pragmatic trials. The TREC program was framed originally and continues to be, using the Promoting Action on Research Implementation in Health Services (PARiHS) framework. Each of our 5 papers will report on a critical aspect of the findings. The symposium will conclude with a discussion of implications of these findings of phase one and a description of phase two and how it is shaped by our interactions with expert panels (context and implementation experts, policymakers, NH managers, paid care providers, and residents and families). Early implications of this work for future research in nursing homes and for practice and policy will be mentioned.

